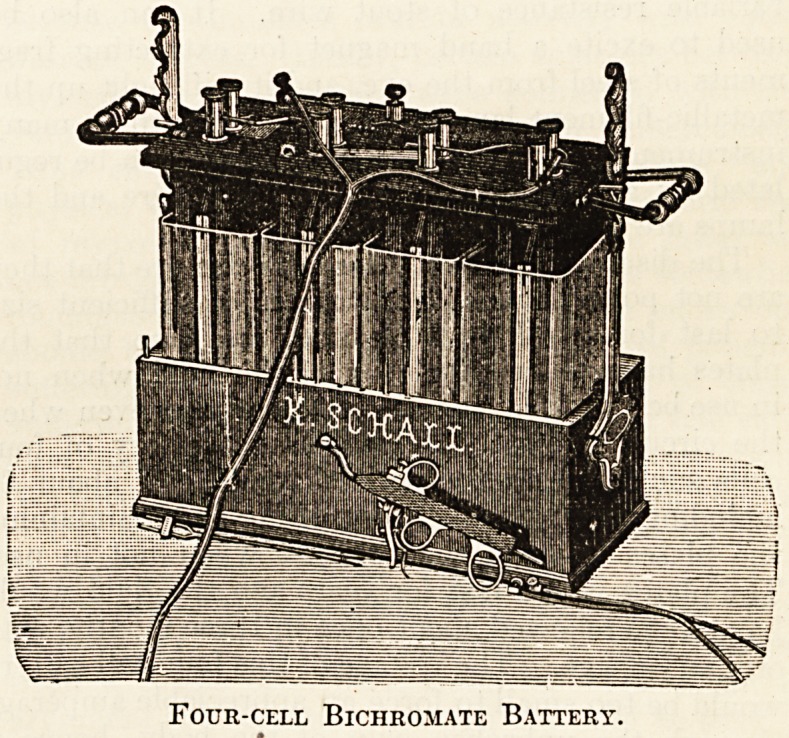# Electricity in Modern Medicine

**Published:** 1911-11-25

**Authors:** Alfred C. Norman

**Affiliations:** House Surgeon, Sunderland and Durham County Eye Infirmary; late House Surgeon in charge of *x.*Ray Department, Royal Buckinghamshire Hospital.


### ELECTRICITY IN MODERN MEDICINE.

### II.—Sources of Electricity.

By ALFRED C. NORMAN, M.D. Edin., House Surgeon, Sunderland and Durham County Eye Infirmary; late House Surgeon in charge of *x*.Ray Department, Royal Buckinghamshire Hospital.

Of the sources of electricity which may be used in medicine some are excellent for certain purposes but their sphere of usefulness is limited, while others can be adapted easily and economically to fulfil practically all medical requirements. At present we shall confine ourselves to the consideration of voltaic or galvanic electricity, that is to say, electricity flowing or tending to flow in a uni-directional current. The most important sources of galvanic electricity are the following: (i) Primary batteries; (ii) accumulators (storage batteries); (iii) the street mains; (iv) private generating plants.

(i) Primary batteries. All chemical action produces some electricity and the object of a primary battery is to utilise chemical action so as to obtain a maximum current at a minimum expenditure of chemicals and labour.

If we partly immerse a plate of zinc and one of copper in an exciting fluid, such as a dilute solution of sulphuric acid, we set up a condition of chemical activity as a result of which there is produced a difference of electrical pressure or potential between the two plates. For want of better terms we say that one plate becomes positively and the other negatively charged with electricity and if we connect the *terminals* of the plates (*i.e.* the parts projecting above the surface of the fluid) by means of a conducting wire we shall get a flow of electricity from copper to zinc through the wire and from zinc to copper through the fluid in the cell. It is with the circuit outside the cell that we have to deal in practice. The zinc terminal in this external circuit is always negative and the current always flows from the positive (+) terminal to the negative (−) terminal.

The arrangement described above is known as a galvanic cell. When we close the circuit of such a cell current flows briskly for a few seconds and then gradually becomes weaker and weaker. This is due to the copper plate becoming covered with bubbles of hydrogen, which gas offers a high resistance to the flow of electricity. The condition is called polarisation and an efficient cell always contains, in addition to the exciting fluid, a chemical "depolariser" capable of absorbing the hydrogen.

The total amount of current (ampèrage) which can be discharged by a cell depends upon the size of the plates and the quantity of exciting fluid, but the pressure at which it is given off—*i.e.* the electromotive force or voltage—depends upon the intensity of the chemical action, and this may be varied by selecting different metals and different exciting fluids. In practice it is impossible to increase the pressure in a single cell beyond two volts, and if we require a higher voltage than this we must obtain a number of cells and connect them *in series* to form a battery, that is to say, we join the negative terminal of the first cell to the positive terminal of the second, the negative of the second to the positive of the third, and so on. We then use the free + terminal of the first cell and the free − terminal of the last cell as the terminals of our battery.

By connecting cells thus in series we add their individual voltages together so that a battery of ten cells each with a voltage of 1.5 would discharge its current at a pressure of 15 volts. This battery on short circuit would not be able to generate a greater total ampèrage than a single cell, but its advantage would lie in the fact that it could drive its ampèrage through a ten times greater resistance in the external circuit.

If we require a greater ampèrage we must obtain larger cells or we may join up our small cells in another way technically known as "in parallel." This consists in connecting all the positive terminals to one wire and all the negative terminals to another wire, these two wires then becoming the + and − terminals of the battery. If we connect up ten small cells in this way the battery would be equivalent to one large cell having plates big enough to give out ten times the ampèrage of a small cell, but the voltage of the battery would remain the same as the voltage of a single cell.

Four-cell Bichromate Battery.

Consequently when we come to construct a battery for any purpose we must first find out how many ampères will be required to do the work in question and we next determine what is the approximate resistance through which that work will have to be done. We can then obtain cells large enough to give out the required ampèrage and connect a sufficient number of them in series to give the voltage necessary to force that current through the estimated resistance.

For instance, in electro-therapeutics we seldom require to send more than one-twentieth of an ampère through any part of the body; consequently in arranging a battery for galvanic treatment we can secure sufficient current from comparatively small cells; on the other hand, the resistance of the skin is very high, 2,500 ohms perhaps, and we shall therefore require considerable voltage to force even one-twentieth of an ampere through it. So we make up our battery of small cells arranged in series and we use from six to forty of these to give what voltage we want for any particular method of application.

There are two well-known batteries which we can use to advantage in medical practice—the bi-chromate and the Leclanché.

Bichromate Cells.—The plates in a bichromate cell are made of zinc and carbon respectively; the exciting fluid is composed of potassium bichromate ½ lb., dissolved in water 5 lb., to which has been slowly added 1¼lb. of strong sulphuric acid.

In country districts where there are no facilities for charging accumulators a medical man could do much useful work with a battery of four large bichromate cells connected in series, producing a total pressure of nearly eight volts (1.95 for each cell). Such a battery is eminently suitable for working a galvano-cautery when controlled by a variable resistance of stout wire. It can also be used to excite a hand magnet for extracting fragments of steel from the eye, and it will light up the metallic-filament lamps now supplied with so many instruments, provided that the current can be regulated by a variable resistance of fine wire and the lamps are not run for long periods.

The disadvantages of these batteries are that they are not portable when constructed of sufficient size to last for any length of time, and also that the plates have to be lifted out of the fluid when not in use because the acid dissolves the zinc even when the circuit is open. A bichromate battery of four cells such as I have described would cost about £4 including an arrangement for lifting and maintaining the plates clear of the solution when not in use. The plates would last for years and the fluid would have to be renewed about once in three months with ordinary usage. The voltage of this four-cell battery would be too small to force an appreciable ampèrage through the unbroken skin of the body, hence it could not be used for most purposes of galvanic treatment.

Leclanche Cells.—The elements of a Leclanché cell are a carbon plate and a zinc rod; the depolariser consists of a mixture of carbon and oxide of manganese packed round the carbon plate in a porous pot, and the exciting fluid is a solution of sal ammoniac, 3 oz. in a pint of water. The electro-motive force of this cell is about 1.5 volt; it is the cell most frequently used in batteries for ringing door-bells because the exciting fluid does not act upon the elements when the circuit is open. A Leclanché cell polarises rather quickly if used for moderately large currents, but it soon depolarises itself when the current is turned off.

Since an ampère is far too much current ever to pass through the human body, the unit of galvanic electricity has been fixed as the milliampère, which is the thousandth part of an ampère.

Now if we wish to destroy a hair follicle by electrolysis a current of two milliampères (*i.e.* the five hundredth part of an ampère) will be quite enough, and a battery giving eight volts would force this current through the partly penetrated epidermis. A six-cell battery costing one guinea would meet our requirements.

In the treatment of facial paralysis we require a current of from six to eight milliampères (see paragraph dealing with current density). This could be furnished by a battery of twelve to eighteen cells which would give us from eighteen to twenty-four volts with which to overcome the resistance of the skin. Such a battery would cost about £2 2s.

For electro-diagnosis and for the treatment of deeply seated nerve lesions we may require a current of eighteen milliampères. For this we should provide a battery of forty cells having a total voltage of nearly sixty; such a battery would cost about £5. It could also be used for the application of most forms of ionic medication.

A few large dry cells are frequently used to make a portable battery for lighting up the small lamps used in medical and surgical instruments.

The technique of electro-therapeutics can be easily read up from a text-book on the subject but it might be well to here mention a few practical details:—

The amount of current a patient is receiving can be roughly estimated from the total voltage of the cells in use but it is a great advantage to measure it accurately by means of a galvanometer (milliampère metre). We can regulate it by means of an arrangement, supplied with nearly all batteries, known as a cell collector. This simply increases or diminishes the voltage between the battery terminals by putting more or less cells into the circuit. In applying the current we connect the active electrode to the positive terminal for some purposes and to the negative for others; this electrode must be of sufficient area to prevent the skin from being damaged by too *dense* a current. The indifferent electrode should be large as possible and applied to an unimportant part of the body.

To illustrate the effect on the tissues of current *density* we might observe that a current of two milliampères applied to the skin from an electrode two inches square would hardly be felt, while the same current applied from the end of a needle would cause pain and produce necrosis of the skin at the point of entrance. This latter fact is utilised in the destruction by electrolysis of a hair follicle or a nævus. Current density may be expressed by the number of milliampères passing through a square centimetre of skin and a safe electrode to use for the currents generally employed in electro-therapeutics would be one with a surface area of three square centimetres.

In concluding this section it would be well to again emphasise that the electrical effects produced on skin, muscle, nerve, etc., are due to ampèrage and ampèrage alone and that the reason why a battery of high voltage will give a painful shock while a much larger one of low voltage can scarcely be felt is because the high voltage battery can force more ampères through the resistance of the body.

(*To be continued.*)

## Figures and Tables

**Figure f1:**